# Supplementary material for: COVID-19 self-testing: Countries accelerating policies ahead of WHO guidelines during pandemics, a global consultation
Source: PLOS Glob Public Health. 2024 Mar 18;4(3):e0002369. doi: 10.1371/journal.pgph.0002369 (PMC10947679; doi:10.1371/journal.pgph.0002369)
Supplement: Attachment — Submitted filename: Response to Reviewers.docx [file pgph.0002369.s001.docx]

Dear PLOS Global Public Health Editor and reviewing team,

Thank you so much for your review and comments and the opportunity to revise and resubmit this manuscript.

We have reviewed your comments carefully and incorporated these into the revised manuscript. In the attached email is both a clean and a track changes version of the manuscript.

In addition, we wanted to highlight that the findings in this report are from an official global consultation process for WHO guidelines. While the procedures for the consultation followed rigorous standards, we hope our revisions have provided clarity as to why the ethics approval process was not the same as that required by a traditional research study. Despite these differences, we believe the findings from this process are an important global contribution as they highlight the real views and perspectives of global stakeholders, and therefore should be given full consideration for publication.

Below we have also provided the point-by-point response on the comments provided in the email and in the manuscript body.

**Comment 1:** Major revision is needed to address the gaps in data collection and survey methods - ethical approval, etc. This is an important study and would like to see it published, but please make sure to address critical information gaps within it.

**Response:** Thank you for this comment. We have sought to review and improve the language used to help clarify that the findings reported in this manuscript are from a consultation used to develop WHO guidance. This process is a well-vetted and tested approach that meets global ethical standards. Previous work has been published in recognition of this. For example, please see publication by Schaeffer et al 2023: <https://link.springer.com/article/10.1007/s10461-023-04093-1>

Please see the following revisions:

Line 1, title: COVID-19 self-testing: Countries accelerating policies ahead of WHO guidelines during pandemics, a global consultation.

Line 18-19: *To inform the development of WHO guidance on COVID-19 self-testing, we conducted a global consultation to gather the views and experiences of policy makers, researchers, and implementers worldwide.*

Line 22-23: *The consultation was conducted by disseminating a WHO questionnaire survey through professional networks via email and social media, encouraging onward sharing.*

Line 29-31: *All information was collated and reported according to WHO guideline development standards and practice for global consultations.*

Line 44-47: *Based on this consultation, systems for the regulatory review, policy development and implementation of C19ST appeared to be much more common in HIC when compared to LIC in early 2022, though most respondents indicated self-testing was available to some extent (101 out of 139 countries) in their country.*

Line 49-52: *The challenges and opportunities highlighted by key stakeholders could be valuable to consider as future testing strategies are being set for outbreak-prone diseases.*

Line 77-80: *We conducted a global consultation targeting policy makers, researchers, and implementers to generate an understanding of policies and experiences with SARS-CoV-2 Ag RDTs for self-testing.*

Line 84-86: *We conducted a global consultation as part of the development of WHO guidelines on COVID-19 self-testing using a self-administered electronic questionnaire from 1st-11th February 2022.*

Line 89-94: *The questionnaire was disseminated globally by the WHO to gather responses from a diverse range of participants, including through country and regional office focal points, through social media (e.g. Facebook, twitter, LinkedIn) and professional networks and associations.* *To ensure a wide distribution, we invited participants to engage in the consultation through professional networks via email and social media and encouraged onward sharing.*

Line 96-98: *We developed the survey consultation questionnaire in SurveyMonkey, which included questions relevant to participants' understanding or perceptions of current policy, practice, and progress of implementing COVID-19 AgRDT in their settings.*

Line 108-112: *We asked respondents stakeholders both closed and open-ended questions related to policy and program information concerning the regulation, availability, target population, indications, implementation, benefits, and challenges of COVID-19 self-testing. Respondents Participants answered questions they were able to and not all respondents answered all questions.*

Line 124-140: *The findings reported are from a global consultation which was initiated as part of to inform the development of WHO guidelines on COVID-19 self-testing. As such, it was carried out in accordance with the WHO Handbook for Guideline Development* [*https://apps.who.int/iris/handle/10665/145714*](https://apps.who.int/iris/handle/10665/145714) *and aligns with standards used in previous published WHO consultations* [*https://link.springer.com/article/10.1007/s10461-023-04093-1*](https://link.springer.com/article/10.1007/s10461-023-04093-1)*. The development of all WHO guidelines is overseen by the WHO Guideline Review Committee. While the project was not set up as a formal research study, a strict protocol was followed according to which the purpose of the consultation was explained at the beginning of each interview, including that results would be shared as part of a global decision-making process and may be published. Verbal informed consent was obtained before starting the consultation. All participants were informed that they could stop the interview at any point.*

*This consultation was conducted with a strong commitment to ethical considerations. The cross-sectional survey design used, disseminated globally via online platforms, ensured voluntary participation. The purpose of the consultation was clear to all participants. Some identifiable data was collected; however, participants' anonymity was strictly maintained during data management analysis and reporting; and no individual identities will be disclosed or compromised.*

Line 143-144: *From 1 to 11 February 2022, 844 individuals participated in the consultation across 139 countries.* *Table 1 describes the characteristics of the participants, including their representation across all WHO regions and income classifications.*

Table 1:

| ***Number of participants(n)*** | *831* | *100%* |
| --- | --- | --- |
| ***WHO Region of Participant’s Country*** | |  |
| *African* | *229* | *27.2%* |
| *Americas* | *80* | *9.5%* |
| *Eastern Mediterranean* | *64* | *7.6%* |
| *European* | *176* | *20.9%* |
| *South-East Asian* | *120* | *14.3%* |
| *Western Pacific* | *173* | *20.5%* |
| ***World Bank Income Classification of Country*** |  |  |
| *Low income* | *74* | *9%* |
| *Lower middle income* | *424* | *51%* |
| *Upper middle income* | *134* | *16%* |
| *High income* | *199* | *24%* |
| ***Level Of Involvement in the COVID-19 Response*** |  |  |
| *National level* | *390* | *47%* |
| *Regional/provincial/District level* | *223* | *27%* |
| *Community level* | *135* | *16%* |
| *Other* | *83* | *10%* |
| ***Affiliation*** |  |  |
| *Academic* | *85* | *10%* |
| *Government* | *378* | *45%* |
| *NGO* | *167* | *20%* |
| *Private sector* | *92* | *11%* |
| *Other* | *109* | *13%* |

***Table 1. Characteristics of consultation participants***

Line 149-150: Out of all participants, 45% identified as government officials, and 47% reported involvement in implementing SARS-CoV-2 activities at the national level.

Line 152-154: *Table 2 outlines the availability of C19ST regulatory processes, policy, and use, as reported by 294 participants from 92 countries across all regions at the time of the survey (early Feb 2022).*

Line 157-158: *Most respondents participants indicated that their countries had a regulatory process in place for reviewing and approving C19ST kits.*

Throughout the manuscript we have sought to emphasize the findings are from a global consultation. Thus, we have changed terminology from “study” and “respondents” to “consultation” and “respondents” or “stakeholders”. Changes to this effect were made, for example in the following areas:

Line 160-165: *Notably, a lack of policy was reported more frequently by participants from low and middle-income countries (LMIC) (25.2%) than by those from high-income countries (HIC) (10.1%). Participants from HIC were more likely to report that C19ST was used in their country compared to those from LMIC (88% vs 19%).*

Line 167-172: *251 participants from 15 countries reported targeting four specific populations for COVID-19 self-testing. Among participants, who indicated that there was a range of different populations noted as being targeted for self-testing, reported that this included testing symptomatic individuals, as well as asymptomatic testing in workplaces, educational institutions and to support attendance at larger gatherings.*

Line 221-224: *This global consultation assessed the extent of policy and implementation of COVID-19 self-testing according to perceptions of targeted policy makers, implementers, and researchers contributing to various levels of COVID-19 response as of early 2022.*
**Comment 2:** Does this manuscript meet PLOS Global Public Health’s [publication criteria](https://journals.plos.org/globalpublichealth/s/criteria-for-publication)? Is the manuscript technically sound, and do the data support the conclusions? The manuscript must describe methodologically and ethically rigorous research with conclusions that are appropriately drawn based on the data presented.

Reviewer #1: No

**Response:** We understand the questions raised and believe we have addressed the concerns about ethics approval by clarifying that this was a global consultation that meets ethical standards and guideline development procedures set by WHO. We have also included reference to similar published consultations such as those by [Schaeffer et al 2023](https://link.springer.com/article/10.1007/s10461-023-04093-1). In the comments above we believe we have addressed these concerns sufficiently and clarified any confusion.

**Comment 3:** How Verbal informed consent was obtained via this online survey. Please explain how and in what context was this consent obtained. This manuscript is very descriptive in nature and designed for a policy decision for a WHO working committee and not intended to be devised as a research study. There were no ethical committee approval obtained for this survey to be claimed as a research study.

**Response:** Thank you for this comment. We have addressed this comment clarifying we were referring to written consent not verbal consent to participate. We have also clarified that the report is that of a consultation and not a traditional research study and survey. Above we have detailed changes to the manuscript to reflect this throughout the paper. We have also referred to similar publications like this one which document the findings from global consultations.
